# Supplementary material for: MetaBAT, an efficient tool for accurately reconstructing single genomes from complex microbial communities
Source: PeerJ. 2015 Aug 27;3:e1165. doi: 10.7717/peerj.1165 (PMC4556158; doi:10.7717/peerj.1165)
Supplement: Supplemental Information 1 [file peerj-03-1165-s001.docx]

**Supplementary Materials**

***Choosing a minimum contig size for robust binning***

As metagenome assemblies contain many small contigs, whether or not to include them is a dilemma: including small contigs will likely improve the genome completeness, at a cost in genome quality because their larger abundance variations make it harder to bin them correctly. We tried to empirically determine a reasonable contig size cutoff by plotting the ratio of mean and variance from the IMG single genome dataset. Although most genome variances are much larger than means, their ratio becomes stabilized after contig size increases to 2.5kb. So the ideal lower contig size for binning would be 2.5kb, but some experiments show that sizes down to 1.5kb could be recruited when assembly quality is excellent. By default, MetaBAT recruits smaller contigs with minimum 1kb to predefined bins that were built using >2.5kb contigs. This second round of recruitment only occurs if there was sufficient abundance correlation (e.g. requiring a minimum of 10 samples as the default).


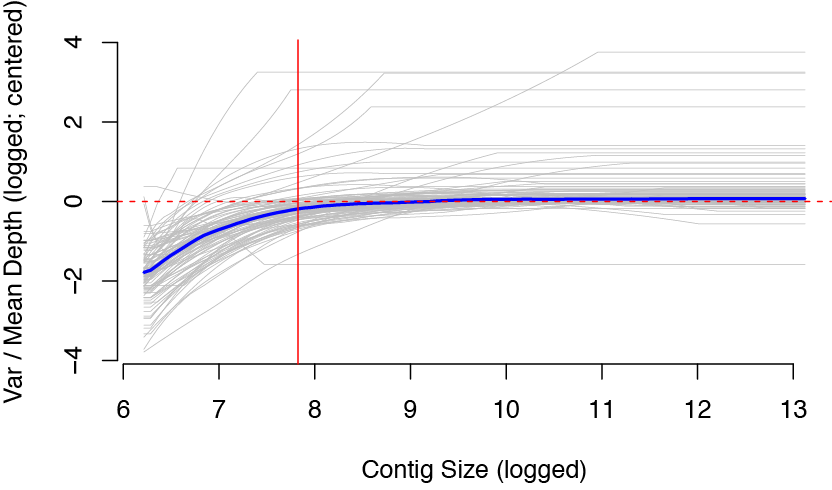


**Supplementary Figure S1.** Choosing a minimum contig size with stable mean variance ratio. The ratio for each individual genome from the IMG dataset was plotted as a gray line, and blue thick line represents the mean ratio of all genomes. 2.5kb (red vertical line) represent a reasonable lower bound of the contig size for a stable mean variance ratio.

***Tetranucleotide Frequency Probability Distance (TDP)***


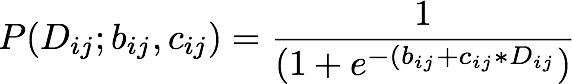


, where *b_ij_* and *c_ij_* are estimated as

***Generation of ‘error-free’ metagenome assembly***

First, we estimated the empirical distribution of metagenome assemblies from MetaHIT data using a truncated exponential distribution having a mean length of 5674 bp. Using the exponential distribution, we generated synthetic metagenome contigs by randomly shredding 290 reference genomes that we found by mapping reads to NCBI bacterial genomes and filtering out >5X genomes (Supplementary Table 1). We consider these contigs as ‘error-free’ contigs so that we could estimate true performance of metagenome binning without being limited by assembly quality. Using 2.5kb lower limit cutoff, we could generate 195601 contigs. Supplementary Figure S2 represents its distribution as a histogram.

**Supplementary Figure S2.** The histogram of simulated contigs, which were drawn from a truncated exponential distribution modeled after real assembly data.

***Evaluation of binning performance***

The three evaluation criteria presented in the main text are defined here. On synthetic data, the recall (completeness) of a genome bin is calculated as the proportion of a reference genome that is covered by a bin’s contigs not counting redundancies. And the precision of a genome bin is the sum of all bases that map to a single reference genome divided by the total bases of that reference. To measure overall quality of a bin, we used F1 as a composite metric of recall and precision. Mathematically they are defined as the following:

For binning evaluation on real assembly data, we used CheckM (Parks et al. 2014). From CheckM output, we used the ‘Completeness’ score as the recall of a genome bin and the inverse of ‘Contamination’ score as the precision. When precision is below 0, we set it as 0. As described in main text, in many cases, most of the contamination in MetaBAT bins derived from cross-assembled contigs, which is reflected in CheckM’s ‘Strain heterogeneity’ measurement. To disregard the strain-level contamination from the precision calculation, we calculated the species level precision as 1 – Contamination * (1 – Strain heterogeneity). This modified species level precision was used to compare genome bins formed by different assemblies from us and from (Nielsen et al.). Supplementary Figure S3 shows the potential benefits from post-processing MetaBAT bins that where aimed to remove cross-assembly strain contamination.

**Binning performance on “error-free” metagenomic assemblies**

**Supplementary Figure S3.** Expanded results of the binning performance on synthetic metagenomic assemblies shown in Figure 3. The number of genomes (X-axis) identified by each binning method (Y-axis) for different recall (completeness) and precision thresholds. Each panel represents the result for a precision threshold.

**Binning performance on real metagenomic assemblies**

**** Supplementary Figure S4.** Expanded results of the binning performance on synthetic metagenomic assemblies shown in Figure 4. The number of genomes (X-axis) identified by each binning method (Y-axis) in different recall (completeness) and precision threshold, which calculates the lack of contamination. Each panel represents the result for a precision threshold.

******

**Supplementary Figure S5.** Comparison between MetaBAT bins and CAG using species level precision after adjusting the precision calculation to only reflect strain-level contamination. Compare it with Figure 5 to notice the changes in both results. Ignoring strain level contamination greatly improved MetaBAT’s precision and F1.

***Post-processing of MetaBAT Genome Bins using Iterative Single Genome Assembly***

The iterative post-processing strategy works as the following:

1. Find a best (e.g. based on coverage) sample for a given bin.

2. Extract reads from the sample that mapped to the bin.

3. Assemble the reads (e.g. velvet).

4. Concatenate the original binned contigs with the latest assembly

5. Run CheckM to assess new assembly

6. Repeat 2-5 until new assembly meet certain criteria.

The above procedures are fairly easy to implement and fast to run. However, more sophisticated approaches can be pursued. For instance, for step 1, we simply picked a sample that had the greatest mean base coverage to a given bin, but it can be extended to search the best target sample. And step 5 can be improved further to run faster since our default choice was to use CheckM, which was not optimized for this procedure. In addition, as shown in Nielsen et al. 2014), other tools can be used to improve draft genome quality such as read error correction, gap closer, or additional sequencing data with longer insert for scaffolding.

***MetaBAT performs better when more samples are supplied***

**Supplementary Figure S6.** Boxplots of the completeness metric (blue) and precision metric (red) of MetaBAT on different number of samples.

**REFERENCES**

Nielsen HB, Almeida M, Juncker AS, Rasmussen S, Li J, Sunagawa S, Plichta DR, Gautier L, Pedersen AG, Le Chatelier E, Pelletier E, Bonde I, Nielsen T, Manichanh C, Arumugam M, Batto JM, Quintanilha Dos Santos MB, Blom N, Borruel N, Burgdorf KS, Boumezbeur F, Casellas F, Dore J, Dworzynski P, Guarner F, Hansen T, Hildebrand F, Kaas RS, Kennedy S, Kristiansen K, Kultima JR, Leonard P, Levenez F, Lund O, Moumen B, Le Paslier D, Pons N, Pedersen O, Prifti E, Qin J, Raes J, Sorensen S, Tap J, Tims S, Ussery DW, Yamada T, Meta HITC, Renault P, Sicheritz-Ponten T, Bork P, Wang J, Brunak S, and Ehrlich SD. 2014. Identification and assembly of genomes and genetic elements in complex metagenomic samples without using reference genomes. *Nat Biotechnol*.

Parks DH, Imelfort M, Skennerton CT, Hugenholtz P, and Tyson GW. 2014. CheckM: assessing the quality of microbial genomes recovered from isolates, single cells, and metagenomes. PeerJ PrePrints.
